# Perforation as a rare presentation of gastric gastrointestinal stromal tumours: a case report and review of the literature

**DOI:** 10.1308/003588414X13824511650010

**Published:** 2014-01

**Authors:** JRA Skipworth, AEE Fanshawe, MJ West, A Al-Bahrani

**Affiliations:** ^1^West Hertfordshire Hospitals NHS Trust,UK; ^2^Fernville Surgery, Hemel Hempstead,UK

**Keywords:** Gastrointestinal stromal tumour, Gastric, Perforation

## Abstract

**INTRODUCTION:**

Gastrointestinal stromal tumours (GISTs) are the most common connective tissue neoplasms of the gastrointestinal tract, the most common clinical presentation of which is with abdominal pain or gastrointestinal bleeding.

**METHODS:**

We describe a case of a perforated gastric GIST as well as reviewing the relevant published literature.

**RESULTS:**

A 51-year-old woman presented to the acute assessment unit with a 1-day history of severe epigastric pain on a background of longstanding reflux symptoms. Radiological investigation demonstrated a perforated mass in the gastric antrum and the patient subsequently underwent an emergency distal gastrectomy. She recovered well postoperatively and was discharged home. Her condition remains stable six months following surgery. Histological analysis revealed the perforated lesion to be a GIST.

A PubMed search suggests that this is the first English report to describe a perforated gastric GIST. Six further published reports (written in English or with an English abstract) describing the presentation of small bowel GISTs with perforation are reviewed.

**CONCLUSIONS:**

We present the first English report of a perforated gastric GIST. More common presentations include abdominal pain and gastrointestinal bleeding. Although rare, GISTs should be considered in the differential diagnoses of perforated gastrointestinal masses.

Gastrointestinal stromal tumours (GISTs) are the most common connective tissue neoplasms of the gastrointestinal (GI) tract, with a worldwide incidence of 11–19.6 per million population, and approximately 700–800 new cases are diagnosed annually in the UK.^1^ GISTs typically affect those over the age of 50 years and have been variably shown to have equal sex incidence or a slight male predominance.^2–4^

GISTs are mesenchymal tumours arising from the muscularis propria of the GI tract and are most commonly diagnosed in the stomach (50%) but they may also arise in the small intestine (25%), colon (10%) or oesophagus (5%), with approximately 10% being found outside of the GI tract.^5–7^ Histologically, GISTs consist of spindle cell, epithelioid or pleomorphic tissue and the majority can be confirmed immunohistochemically via staining for KIT protein (CD117 antigen positive).^8^ However, some tumours have PDGFRA mutations instead, rendering them CD117 negative; in this instance, DOG1 is a useful surrogate marker as it is highly expressed in both typical GISTs and KIT mutation negative GISTs.^9^ Generally, GISTs are benign in 70–80% of cases, particularly those located in the stomach, where benign tumours are 3–5 times more common than malignant.^2,8^

The most common clinical presentation of a GIST is with abdominal pain or GI bleeding, which manifests either chronically as anaemia or acutely as melaena or haematemesis. Other presentations include bloating and, less commonly, intestinal obstruction, nausea, weight loss and a palpable mass.^3,10^ Delayed presentation can also occur: GISTs under 2cm are often asymptomatic and when symptoms do occur, they tend to be non-specific and 50% of malignant GISTs are therefore metastatic at the time of diagnosis.^7,11^

We describe a case of a gastric GIST presenting with a perforation and review the relevant published literature.

## Methods

An online PubMed search was performed to identify all reports of perforation associated with GIST using the search terms ‘perforated’, ‘perforation’, ‘acute abdomen’, ‘gastrointestinal stromal tumour’ and ‘GIST’ in various combinations. All relevant articles written in English (or with an English abstract containing pertinent information) were reviewed. Six other relevant reports were identified and are reviewed above (Table 1).^12–17^

**Table 1:** Summary of case reports describing perforated gastrointestinal stromal tumours

Case report	Age/sex	Past medical history	Presentation	Operative management	Diagnosis/anatomic site	Malignant potential	Outcome
Present case	51F	Reflux symptoms	Abdominal pain → ultrasonography showed inflammation → CT → perforated mass in gastric antrum	Laparotomy: distal gastrectomy with Roux-en-Y retrocolic gastrojejunostomy	Perforated 5cm GIST arising from gastric antrum	Low	Well (6 months following surgery); treated with imatinib
Mitura, 2012^12^	63F	Nil	Hypogastric abdominal pain and fever → outpatient ultrasonography → hypogastric tumour	Laparotomy: segmental ileal resection	Perforated 14cm GIST arising from ileal Meckel’s diverticulum	High	Well with no disease recurrence (6 months following surgery); no chemotherapy given
Chou, 2011^13^	76F	Nil	Lower abdominal cramping pain → CT → intraperitoneal free air and distended diverticulum	Laparoscopy: segmental ileal resection	Perforated 3.2cm GIST arising from ileal Meckel’s diverticulum	High	Unknown
Dogrul, 2010^14^	86F	Hypertension, coronary artery disease, cholecystectomy, total hip replacement, TAH and BSO	Abdominal pain, nausea and vomiting → CT → ileal perforation, with dilation and oedema of proximal ileum	Laparotomy: 20cm small bowel resection with end-to-end anastomosis; re-exploration on day 7 due to anastomotic leak	Perforated 8cm GIST arising from ileal Meckel’s diverticulum	High	Died 2 months following surgery from sepsis/multiorgan failure
Hur, 2008^15^	70M	Previous high risk gastric GIST (1993), recurrence in gastrohepatic ligament (2001), hepatic recurrence (2002)	Patient on chemotherapy (sunitinib) at time of presentation with diffuse abdominal pain → CT → necrosis of recurrent hepatic mass and perforation of invaded transverse colon	Hepatic recurrence not resected due to poor patient baseline; percutaneous drainage of intraperitoneal pus, with antibiotic treatment	Perforated hepatic/colonic recurrence	High	Well on chemotherapy, with stable disease (after completion of second cycle of sunitinib)
Efremidou, 2006^16^	66M	Two previous episodes of upper gastrointestinal haemorrhage (managed conservatively)	Diffuse abdominal pain, vomiting and abdominal distension (no abnormalities on CXR, AXR or ultrasonography)	Laparotomy: 13cm ileal resection and regional lymph node excision	Perforated 7cm GIST arising from ileum	Intermediate	Well with no disease recurrence (44 months following surgery); chemotherapy (imatinib) given for first 20 months
Szentpáli, 2004^17^	70M	Type 2 diabetes mellitus, cerebrovascular disease, myocardial infarction, hypertension	Right lower abdominal pain → ultrasonography → thick and hypervascularised bowel wall	Laparotomy: 15cm small bowel resection with side-to-side anastomosis	Perforated 1.5cm GIST arising from small bowel Meckel’s diverticulum	‘Borderline’ (small tumour size, low mitotic index but mucosal invasion)	Well with no disease recurrence (3 years following surgery)

CT = computed tomography; GIST = gastrointestinal stromal tumour; TAH = total abdominal hysterectomy; BSO = bilateral salpingo-oophorectomy; CXR = chest x-ray; AXR = abdominal x-ray

Other additional reports were excluded due to lack of information or foreign language content only. These included two reports of gastric GIST perforation,^18,19^ a perforated duodenal GIST in the context of neurofibromatosis type 1,^20^ a perforated small bowel GIST^21^ and a perforated GIST associated with a Meckel’s diverticulum.^22^

## Case history

A 51-year-old woman presented to the acute assessment unit at Watford General Hospital, an oesophagogastric cancer centre, with a 1-day history of sudden onset, severe, epigastric pain. Her past medical history included reflux symptoms treated with antacids and omeprazole by her general practitioner as well as menopausal symptoms treated with clonidine. She had no recent use of non-steroidal anti-inflammatory drugs or steroids and had no family history of note. Alcohol intake was limited and she was an ex-smoker. Further history revealed that she had noticed no weight loss but she did report ‘black, sticky’ stools in the year prior to admission.

Initial assessment demonstrated that the patient was tachycardic (109bpm) with a blood pressure of 108/67mmHg. She was afebrile and saturating normally without supplemental oxygen. Abdominal palpation revealed a soft but distended abdomen, with tenderness in the epigastrium. Digital examination of the rectum was normal. Venous blood tests revealed a haemoglobin level of 10.5g/dl (normocytic) and a C-reactive protein level of 165mg/l. The white cell count, liver function tests, renal function and electrolytes were normal. Erect chest radiography demonstrated no free air under the diaphragm but there was a prominent gastric bubble. Assessment of arterial blood gases and plain abdominal radiography was unremarkable.

**Figure 1:**
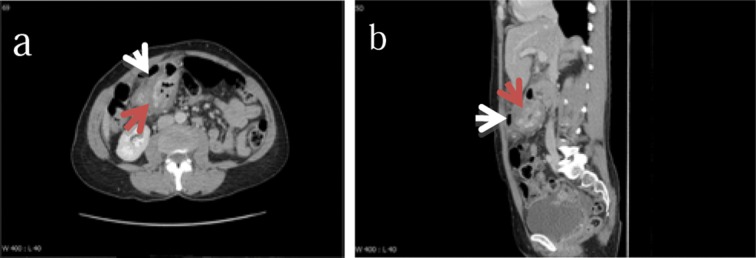
Axial and sagittal contrast enhanced computed tomography of a thickened and heterogeneously enhancing mass in the gastric antrum (dark arrow) with surrounding soft tissue stranding and localised free air anterior to the mass (light arrow)

Abdominal ultrasonography was organised, showing an irregular thickening in the right side of the epigastrium, suggestive of an inflamed duodenum with surrounding inflammatory changes. Urgent computed tomography (CT) of the abdomen and pelvis was therefore arranged, demonstrating a markedly thickened and heterogeneously enhancing mass in the gastric antrum, extending into the duodenum and associated with localised perforation and surrounding soft tissue stranding (Fig 1).

The patient proceeded to an urgent laparotomy, at which a 5.5cm perforated antral tumour was found with minimal contamination and no evidence of peritoneal or liver metastases. A distal gastrectomy with a Roux-en-Y retrocolic gastrojejunostomy was performed. The patient made an uneventful recovery on the ward, and a barium swallow test on day 6 demonstrated no contrast leak and good flow through the anastomosis. She was discharged on the ninth postoperative day.

Subsequent histopathology revealed the tumour to be a mixed cell type GIST (CD117 negative, DOG1 positive) demonstrating mucosal invasion and ulceration but no haemorrhage or necrosis. There was transmural infiltration through the mucosa and into the serosa but tumour margins were clear. There was no lymphovascular invasion and all of the 17 sampled lymph nodes were tumour free. Based on tumour size (maximum diameter 5.5cm) and mitotic index (<1/5mm^2^), the tumour was classified as low risk for progressive disease (3.6%) on Miettinen’s criteria.^23,24^ However, although histological assessment may define a low risk of progressive disease, novel criteria suggest that perforation may have an adverse effect on relapse free survival and the risk of progression may therefore be as high as 40–60% on Joensuu criteria.^25^

At the outpatient follow-up appointment, abdominal examination was unremarkable and the patient continued to recover well. She was discussed at the upper GI multidisciplinary meeting and referred to oncology colleagues for consideration of further treatment with tyrosine kinase inhibitors as well as being entered into a surveillance programme comprising three-monthly clinical reviews and six-monthly CT. Following the oncology review, she was commenced on empirical imatinib (400mg once daily) and remains well at six months following surgery. However, at her most recent oncology review, results of genetic analysis demonstrated that the tumour had no mutations of KIT or PDGFRA, thereby confirming a wild type tumour. European Society for Medical Oncology guidelines contain no current consensus regarding the efficacy of imatinib use in such wild type tumours and the patient is therefore awaiting further discussion regarding whether to continue with current treatment.

## Literature review

Our literature search revealed that perforation of GISTs occurs in an older population group (all >50 years), with equal sex incidence (Table 1). All patients presented with abdominal pain. Four of the reports demonstrated GIST perforation occurring in the context of a Meckel’s diverticulum,^12–14,17^ one report described a perforation of the transverse colon following local invasion by a hepatic GIST recurrence^15^ and one described a GIST perforation in the small intestine.^16^ In five of the six cases, small bowel resection was performed with primary anastomosis.^12–14,16,17^ In the case of hepatic recurrence with local invasion, surgical management was not appropriate due to the patient’s poor baseline status.^15^ Tumour size was variable, ranging from 1.5cm to 14cm. In four of the six cases, malignant potential of the GIST was high.^12–15^ Despite perforation (and in some cases, significant co-morbidity), four of the six cases reported good outcomes.^12,15–17^

## Discussion

### Presentation

The majority of reports described perforated small intestinal GIST; whereas, we report the only case of a perforated gastric GIST, despite a higher overall incidence of GISTs at this anatomical site.^5–7^ The reasons underlying this low gastric perforation rate remain unclear but may include a thicker gastric wall compared with the small and large bowel, enhanced awareness and recognition of gastric/upper GI symptoms, as well as greater ease of investigation via an oesophagogastroduodenoscopy, and subsequent earlier investigation and diagnosis; as compared with non-specific small intestinal symptoms with more problematic investigative methods. Furthermore, the higher incidence of benign disease in gastric GISTs may lead to a more indolent progression and lower likelihood of perforation.

In the case described in this paper, the patient had longstanding symptoms of upper GI pathology (reflux, melaena) and should have undergone investigation at an earlier stage. This could potentially have avoided emergency presentation and operation.

### Malignant potential

Most of the previously published reports describe perforated GISTs of high malignant potential, unlike the report described in this manuscript, which involves a GIST of low malignant potential. Whereas malignant GISTs are more likely to have metastasised, the effect of malignant potential on symptom profile is not well described and it remains unclear whether they are more likely to present with perforation.^5^ The tumour described in our report was histochemically unlike the majority of GISTs in that it expressed DOG1 rather than CD117. Such CD117 negative GISTs have been shown to be of mostly low or no malignant potential^26^ and the presentation of such a low risk GIST with perforation may therefore be secondary to investigative delay rather than histological profile.

### Management

The management of the perforated GISTs described above was via emergency surgical resection (five laparotomies, one laparoscopy) in all but one case, where the patient was felt to be too high risk for surgical intervention. Surgical resection is also the mainstay of treatment for localised GISTs presenting non-acutely.^3^ Further treatment options are based on prognostic factors including tumour size and mitotic rate, and include adjuvant chemotherapy in the form of imatinib, a tyrosine kinase inhibitor, which results in prolonged survival rates in advanced or metastatic disease when used as a primary treatment.^27,28^ In cases of disease progression on imatinib, a second-line tyrosine kinase inhibitor, sunitinib, may also be used.^29^

### Survival after perforation

The mortality rate after spontaneous perforation of the small bowel or stomach from any cause remains high (>25%).^30–32^ Five of the seven patients discussed here were described as well following emergency surgery (with only one having died from anastomotic leak and subsequent multiorgan failure), demonstrating that expeditious resuscitation and surgical management remains the mainstay of treatment in all cases of perforation.

## Conclusions

This case is the first English report of a gastric GIST presenting with perforation and an acute abdomen. Although rare, GISTs should be considered as a differential diagnosis of gastric and other GI masses that present with perforation. Our case further demonstrates the need for prompt recognition and appropriate investigation of new onset upper GI symptoms in both older and younger age groups to exclude potentially sinister causes.
